# Plant-based dietary pattern and low muscle mass: a nation-wide cohort analysis of Chinese older adults

**DOI:** 10.1186/s12877-023-04265-7

**Published:** 2023-09-16

**Authors:** Longbing Ren, Yuhong Tang, Rui Yang, Yang Hu, Jingjing Wang, Shaojie Li, Mingzhi Yu, Yuling Jiang, Zhouwei Liu, Yifei Wu, Ziqi Dong, Yi Zeng, Faqin Lv, Yao Yao

**Affiliations:** 1 China Center for Health Developments, Peking University, Beijing, China; 2 School of Medicine, Tongji University, Shanghai, China; 3 Center for Healthy Aging and Development Studies, National School of Development, Peking University, Beijing, China; 4 Center for Study of Aging and Human Development and Geriatrics Division, School of Medicine, Duke University, Durham, U.S.A.; 5 Ultrasonic Department, The Third Medical Center of Chinese People’s Liberation, Army General Hospital, Beijing, China; 6 Key Laboratory of Epidemiology of Major Diseases (Peking University), Ministry of Education, Beijing, China

**Keywords:** Plant-based dietary pattern, Animal-based food, Muscle mass, Sarcopenia prevention, Older people, Functional dependency

## Abstract

**Background:**

It remains unclear whether plant-based or animal-based dietary patterns are more beneficial for older adults more in maintaining muscle mass. Using a prospective cohort with nationwide sample of China older adults in this study, we aimed to examine the relationship between adhering to plant-based diet patterns or animal-based diet patterns and muscle loss.

**Methods:**

We included 2771 older adults (≥ 65 years) from the Chinese Longitudinal Health Longevity Survey (CLHLS) with normal muscle mass at baseline (2011 and 2014 waves), which followed up into 2018. Plant-based dietary pattern scores and preference subgroups were constructed using 16 common animal-based and plant-based food frequencies. We used the corrected appendicular skeletal muscle mass (ASM) prediction formula to assess muscle mass. We applied the Cox proportional hazard risk regression to explore associations between dietary patterns and low muscle mass (LMM).

**Results:**

During a mean of 4.1 years follow-up, 234 (8.4%) participants with normal muscle mass at baseline showed LMM. The plant-based dietary pattern reduced the risk of LMM by 5% (Hazard Ratios [HR]: 0.95, 95% confidence intervals [95%CI]: 0.92–0.97). In addition, a high plant-based food company with a high animal-based food intake pattern reduced the risk of LMM by 60% (HR: 0.40, 95% CI: 0.240–0.661) and 73% (HR: 0.27, 95% CI: 0.11–0.61) in the BADL disability and IADL disability population compared with a low plant-based food and high animal-based food intake, whereas a high plant-based food and low animal-based food intake was more beneficial in reducing the risk of LMM in the normal BADL functioning (HR: 0.57, 95% CI: 0.35–0.90) and IADL functioning (HR: 0.51, 95% CI: 0.28–0.91) population.

**Conclusions:**

When it comes to maintaining muscle mass in older Chinese people with functional independence, a plant-based diet pattern is more beneficial and effective than the animal-based one. People with functional dependence may profit from a combination of plant-based and animal-based diets to minimize muscle loss.

**Supplementary Information:**

The online version contains supplementary material available at 10.1186/s12877-023-04265-7.

## Introduction

Sarcopenia, an age-related disease, manifests as an accelerated low muscle mass (LMM) and function (LMF) and is strongly associated with subsequent falls, disability, and death [1]. With increasing global aging, sarcopenia has become one of the dominant health challenges for older adults. At the same time, patients with sarcopenia have higher medical needs and health-related costs, which impose a heavy medical and economic burden on individuals and society, especially in low- and middle-income countries (LMICs) [2]. However, no specific drugs have been approved for the medication of sarcopenia, and exercise combined with nutritional interventions remains the dominant means of maintaining muscle mass [1, 3].

Despite the importance of adequate protein intake in maintaining muscle mass, the optimal source of protein for older adults remains controversial. Serval previous studies have demonstrated that animal protein, which has a greater absorption rate and better quality, has significant benefits in maintaining muscle mass in older adults [4, 5]. Research from Japan found a positive correlation between muscle mass and animal protein consumption in older women over 75 [6]. However, results from a cross-sectional study from mainland China, indicated that muscle mass was associated with plant protein intake and total protein intake, rather the animal-based protein intake [7]. This study revealed that plant-based foods may play a core part in maintaining muscle mass.

The importance of dietary intake is not solely based on nutrient intake but on deeper consideration of the impact of overall dietary patterns on human health. A recent study of 347 middle-aged Australians found that a plant-based diet was positively associated with limb muscle strength [8]. A plant-based diet meets the protein requirement of older adults with simultaneously rich antioxidants and anti-inflammatory nutrients that can help mitigate LMM by reducing chronic inflammation and oxidative stress, also the risk of muscle loss in older adults [1, 9–11].

However, research on plant-based dietary patterns and muscle mass in older adults is still lacking, especially for LMICs. The variability in the richness of the research content is unsurprising, as it has been shown that dietary culture and nutritional intake levels receive the influence of cultural and socioeconomic factors [12, 13]. On the other hand, over 80% of the world's older adults will live in LMICs in 2050 [14]. Since LMICs often have lower BMI levels and higher malnutrition prevalence than high-income countries, further research is necessary in these countries [15]. Research on dietary patterns and LMM among older adults in LMICs has mostly been cross-sectional, with inadequate analysis of confounding and causation. Longitudinal studies are therefore urgently required with greater levels of evidence [16].

To fill the research gap, we conducted a prospective longitudinal study with a nationwide sample of older people to examine the relationship between adhering to plant-based diet patterns or animal-based diet patterns and muscle loss. Our study may help guide the dietary recommendations for reducing the risk of muscle loss in older groups from LMICs.

## Materials and methods

### Study population

The China Longitudinal Healthy Longevity Survey (CLHLS) is a prospective cohort study of the Chinese elderly population aged 65 years and older, using a multistage whole-group sampling method to recruit potential participants from 23 of China's 31 provinces, with a sample that can represent approximately 85% of the total Chinese population. The study started in 1998 with follow-up surveys every 3–4 years. A more detailed description of the sampling design can be found elsewhere [17]. All participants signed informed consent at baseline and each follow-up. the CLHLS study was approved by the Biomedical Ethics Committee of Peking University, China (IRB00001052-13074).

**Fig. 1:**
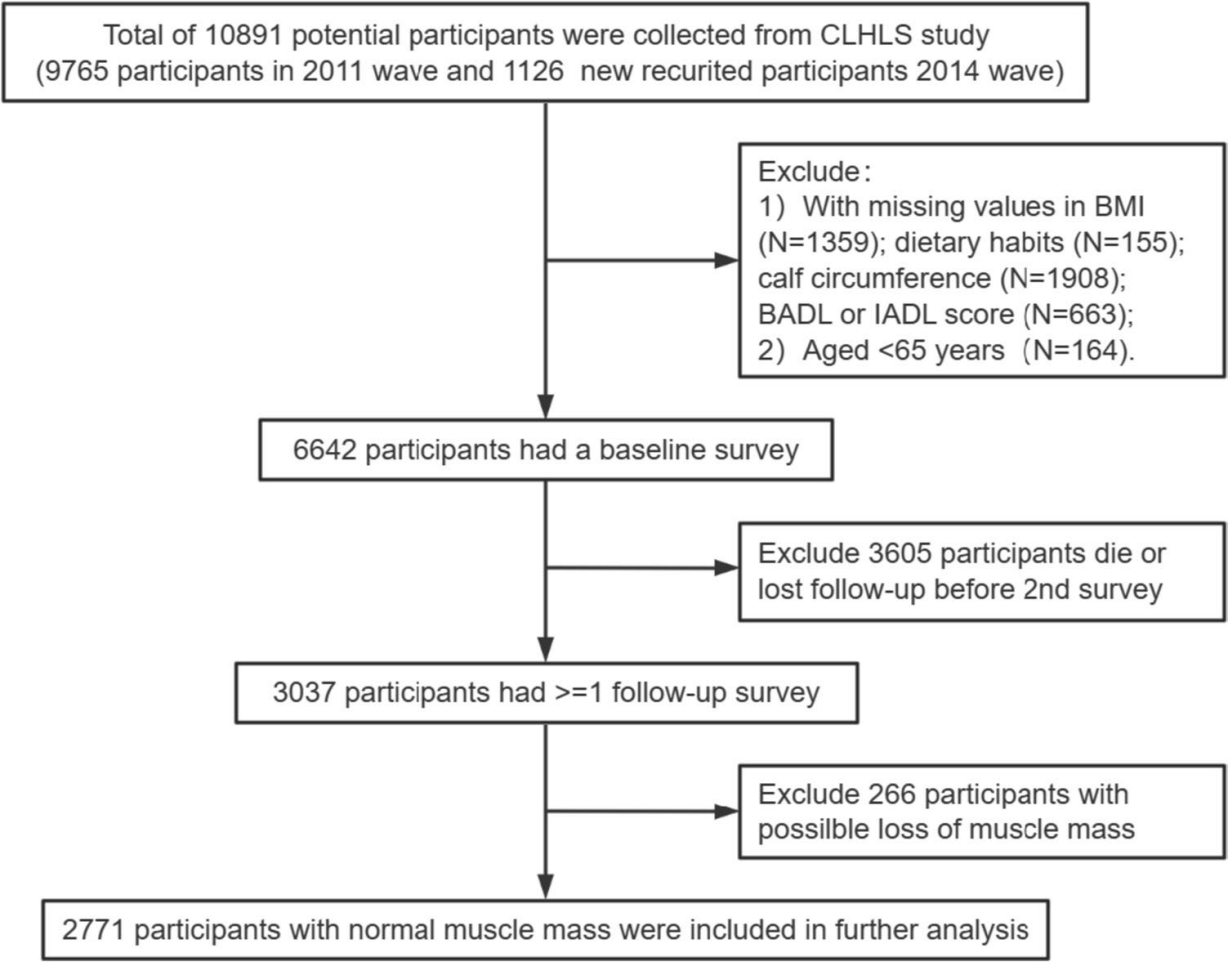
Flow chart of study design

Since calf circumferences were first measured in some areas in the 2011 wave, we recruited people who had their first calf circumference measurement in the 2011 wave and the 2014 wave, and the next one (recruited in the 2014 wave) or two (recruited in the 2011 wave) subsequent survey as follow-up outcomes. We excluded participants who were younger than 65 years old, had missing values in the dietary pattern, BMI, and two ADL scores, and LMM at baseline. In total, 2771 participants were included in the analysis. More details on participant inclusion and exclusion can be found in Fig. 1.

### Assessment of dietary pattern

A food frequency questionnaire was used for the dietary pattern assessment. A total of sixteen food items were included in the study, covering the most common parts of the daily diet among the Chinese population. All the types of food were divided into animal-based and plant-based categories according to their sources. Plant-based foods included: grains, vegetable oils, fresh fruits, fresh vegetables, legumes, garlic, nuts, tea, salt-preserved vegetables, sugar, and mushrooms or algae. Animal-based foods include animal fats, milk and related dairy products, eggs, fish, and meat.

In this study, the non-quantitative form of the questionnaire designed by calculating the frequency of food intake and thus scoring the dietary pattern is reliable and valid, although it lacks detailed quality [18, 19]. Legumes, garlic, nuts, tea, salt-preserved vegetables, sugar, mushrooms and algae, milk and related dairy products, eggs, fish, and meat were recorded in the intake frequency as five categories in CLHLS. The frequency of fruits and fresh vegetables were recorded as four categories. The frequency of animal food such as milk and related dairy products, eggs, fish, and meat were also scored as sugar and salt-preserved vegetables. The plant foods were scored 5 for the most frequent consumption and 1 for the least frequent. More details on constructing and scoring dietary indices can be found in Table S1.

The Plant-based Diet Index (PDI), constructed logically as the sum of the animal-based food score and plant-based food score, is theorized to range from 16 to 80, with higher scores indicating more frequent consumption of plant-based foods.

Considering that variety may cause confusion, e.g., more overall variety and less overall variety may have similar scores, we also ranked the plant-based food scores and animal-based food scores of all participants, respectively, and then divided them into two halves according the median level, finally combined the both of two categories into four new dietary groups: Group 1: low plant food consumption and high animal food consumption; Group 2: low plant food consumption and low animal food consumption; Group 3: high plant food consumption and high animal food consumption; and Group 4: high plant food consumption and low animal food consumption.

### Assessment of possible low muscle mass

Muscle mass was usually defined as measured appendicular skeletal muscle mass (ASM) by dual-energy X-ray absorptiometry (DXA) or bioimpedance method, and calf circumference was often used to screen for possible muscle mass loss in the community according to the Asian Working Group for Sarcopenia [3]. However, because obesity types may affect the accuracy of LMM screening through calf circumference [20], we use an ASM prediction equation calculated by calf circumference, waist circumference, height and weight to calculate ASM, then evaluated LMM [21]. The ASM prediction equation was: ASM (kg) = 2.955 * sex (men = 1, women = 0) + 0.255 * weight (kg)—0.130 * waist circumference (cm) + 0.308 * calf circumference (cm) + 0.081 * height (cm)—11.897 (adjusted R^2^ = 0.94, standard error of the estimate = 1.2 kg). The LMM was defined as < 7.0 kg*m^−2^ in men and < 5.7 kg*m^−2^ in women calculated by ASM*height^−2^.

### Assessment of physical function levels

Physical function levels were assessed by basic activity of daily living (BADL) and instrumental activity of daily living (IADL) questionnaire. BADL was measured with the following six aspects in CLHLS: (1) Bathing; (2) Dressing; (3) Toileting; (4) Indoor moving; (5) Continence of defecation; (6) Eating. IADL was rated with eight questions: (1) Can you visit your neighbors by yourself? (2) Can you go shopping by yourself? (3) Can you cook a meal by yourself when necessary? (4) Can you wash clothes by yourself when necessary? (5) Can you walk a kilometer at a time by yourself? (6) Can you lift a weight of 5 kg, such as a heavy bag of groceries? (7) Can you continuously squat and stand up three times? (8) Can you take public transportation by yourself? Each question has three answers: 1 representing complete independence; 2 representing partial dependence; and 3 representing complete dependence. BADL disability was defined as one or more of the six aspects that cannot be completely independence, and IADL disability was defined as one or more of the eight aspects that cannot be completely independence.

### Covariates

Sociodemographic characteristics, health behavior and history of disease were considered to control the potential bias. Sociodemographic characteristics included age (less than 80 years or not), gender (male or female), residence (city, town, and rural), education (0, 1–6, and ≥ 7 years), annually regular physical examination, hunger in childhood. Annually regular physical examination (yes vs. no) was assessed by asking “Do you have regular physical examination once every year?”. Hunger in childhood (yes vs. no) was assessed by asking “Did you frequently go to bed hungry as a child?”. Health behaviors included physical exercise, smoking, and drinking. Physical exercise (yes vs. no) was measured by asking “do you do exercises regularly at present?”. Smoking (yes vs. no) was assessed by asking “do you smoke at the present time?” and drinking (yes vs. no) was assessed by asking “do you drink alcohol at the present time?”. History of disease was based on self-reported hypertension, diabetes, heart disease, stroke and other cerebrovascular, arthritis and dyslipidemia. Cognitive function was measured by Mini-Mental State Examination (MMSE).

### Statistical analysis

Missing covariates values were filled through multiple imputation methods. Descriptive statistics were used to summarize the baseline characteristics. Multivariate Cox proportional hazard regression was calculated for the hazard ratios (HRs) and 95% confidence intervals (95% CI) for the association between PDI pattern and risks of LMM during the follow-up. We also developed four Cox models to evaluate the robustness of PDI and different dietary groups on LMM. The base model (Model 1) controlled for baseline ASM, gender and age; Model 2 further controlled for residence, hunger in childhood, and education; Model 3 additionally controlled for BADL disability, IADL disability, regular physical examination, current exercise, smoking and drinking, and MMSE score; and Model 4: added history of disease in Model 3. Considering the relatively old age among our participants, a competing risk model was built as a sensitivity analysis for evaluating the bias caused by competing risk from death. We used model 4 further stratified the associations of dietary groups and LMM by physical function levels in order to seek the best dietary pattern for people with physical dysfunction. All statistical analysis was conducted using R software (version: 4.1.2, R Core Team, R Foundation for Statistical Computing, Vienna, Austria). Statistical significance was defined by p < 0.05 in two-sided testing.

## Results

### The characteristics of study participants

A total of 2771 participants with normal muscle mass were enrolled in our study. The baseline characteristics according to different dietary groups are presented in Table 1. Nearly half (49.2%) of participants were over 80 years old, 53.0% were male, 60.6% were rural residents, and 48.9% were without education experience. About 49.0% of participants had regular physical examinations and 78.9% of participants suffered hunger during childhood. For unhealthy choices, such as smoking and alcohol taking, around one-fifth of the participants each have consumption habits, 20.5% and 19.8% respectively. 7.0% of participants were unable to finish basic activities of daily living and 44.6% of participants had IADL disability. Compared to the lowest PDI score group (Group 1), the participants with other groups are more likely to be men, younger, city residents, have higher education levels, have regular physical examination habits, and have more chronic disease.

**Table 1:** Baseline characteristics of participants by different PDI groups

Characteristic	Total (*N* = 2771)	Group 1 (*N* = 561)	Group 2 (*N* = 801)	Group 3 (*N* = 767)	Group 4 (*N* = 642)	*p* Value
LMM during follow-up, %	234 (8.44)	68 (12.12)	92 (11.49)	34 (4.43)	40 (6.23)	< 0.001
PDI, score	48.45 ± 5.74	41.19 ± 3.80	46.92 ± 3.49	50.50 ± 3.54	54.27 ± 3.43	< 0.001
Plant-based food, score	32.75 ± 5.31	28.05 ± 3.29	28.66 ± 2.89	37.37 ± 3.40	36.43 ± 2.99	< 0.001
Animal-based food, score	15.71 ± 3.07	13.14 ± 1.75	18.26 ± 2.04	13.12 ± 1.60	17.84 ± 1.86	< 0.001
Age, %						< 0.001
< 80 years	1,407 (50.78)	248 (44.21)	358 (44.69)	435 (56.71)	366 (57.01)	
≥ 80 years	1,364 (49.22)	313 (55.79)	443 (55.31)	332 (43.29)	276 (42.99)	
Gender, men, %	1,468 (52.98)	300 (53.48)	361 (45.07)	444 (57.89)	363 (56.54)	< 0.001
Education, %						< 0.001
0 year	1,354 (48.86)	297 (52.94)	484 (60.42)	266 (34.68)	307 (47.82)	
1–6 years	783 (28.26)	150 (26.74)	209 (26.09)	236 (30.77)	188 (29.28)	
> 6 years	634 (22.88)	114 (20.32)	108 (13.48)	265 (34.55)	147 (22.90)	
Residence, %						< 0.001
City	318 (11.48)	34 (6.06)	34 (4.24)	176 (22.95)	74 (11.53)	
Town	774 (27.93)	170 (30.30)	223 (27.84)	209 (27.25)	172 (26.79)	
Rural	1,679 (60.59)	357 (63.64)	544 (67.92)	382 (49.80)	396 (61.68)	
Regular physical examination, %	1,359 (49.04)	288 (51.34)	356 (44.44)	420 (54.76)	295 (45.95)	< 0.001
Hunger in childhood, %	2,185 (78.85)	444 (79.14)	661 (82.52)	557 (72.62)	523 (81.46)	< 0.001
Exercise, %	854 (30.82)	127 (22.63)	198 (24.72)	302 (39.37)	227 (35.36)	< 0.001
Smoking, %	569 (20.53)	110 (19.61)	156 (19.48)	172 (22.43)	131 (20.40)	0.470
Drinking, %	548 (19.78)	115 (20.50)	111 (13.86)	187 (24.38)	135 (21.03)	< 0.001
ASM, score	6.26 ± 1.35	6.01 ± 1.18	6.08 ± 1.27	6.52 ± 1.12	6.43 ± 1.23	< 0.001
MMSE, score	26.48 ± 5.42	26.94 ± 4.68	25.04 ± 6.72	27.48 ± 4.02	26.66 ± 5.32	< 0.001
BADL disability, %	193 (6.96)	34 (6.06)	58 (7.24)	56 (7.30)	45 (7.01)	0.816
IADL disability, %	1,232 (44.46)	249 (44.39)	421 (52.56)	299 (38.98)	263 (40.97)	< 0.001
Hypertension, %	941 (33.96)	157 (27.99)	253 (31.59)	299 (38.98)	232 (36.14)	< 0.001
Diabetes, %	140 (5.05)	11 (1.96)	38 (4.74)	60 (7.82)	31 (4.83)	< 0.001
Heart disease, %	325 (11.73)	50 (8.91)	90 (11.24)	103 (13.43)	82 (12.77)	0.063
Stroke, cerebrovascular disease, %	224 (8.08)	33 (5.88)	66 (8.24)	66 (8.60)	59 (9.19)	0.170
Arthritis, %	318 (11.48)	47 (8.38)	99 (12.36)	94 (12.26)	78 (12.15)	0.084
Dyslipidemia, %	119 (4.29)	17 (3.03)	30 (3.75)	47 (6.13)	25 (3.89)	0.026

*Abbreviations*: *PDI* plant-based dietary index; Group 1: low plant food consumption and high animal food consumption; Group 2: low plant food consumption and low animal food consumption; Group 3: high plant food consumption and high animal food consumption; Group 4: high plant food consumption and low animal food consumption; *LMM* low muscle mass, *BADL* basic activity of daily living, *IADL* instrumental activity of daily living, *ASM* appendicular skeletal muscle mass, *MMSE* Mini-Mental State Examination

### Dietary scores and LMM

After an average of 4.11 years of follow-up, 234 of 2771 participants (8.4%) suffered LMM, and the incidence differed across different dietary groups. The baseline characteristics by follow-up LMM are shown in Table S2. The participants who had the higher score of PDI showed lower hazards of having LMM in model 1 controlling for demographic variables (HR = 0.94, 95% CI: 0.92–0.96). After additional socioeconomic factors, health behaviors and history of disease were controlled for in model 2, model 3 and model 4, PDI remained significantly linked to decreased hazards of experiencing LMM by about 5.5 ~ 5.7% (Table 2 & Table S3). Plant-based food scores showed similar results to the PDI, with significant protection against muscle mass in different models (All *p*-value < 0.05), whereas animal-based food scores performed as non-significant in all models (All *p*-value > 0.05).

**Table 2:** Associations of PDI with possible low muscle mass among whole sample

Indicators	Model 1	Model 2	Model 3	Model 4
PDI	0.94 (0.92–0.96) ***	0.94 (0.92–0.97) ***	0.94 (0.92–0.96) ***	0.95 (0.92–0.97) ***
Plant-based food score	0.94 (0.92–0.97) ***	0.95 (0.92–0.97) ***	0.95 (0.96–0.97) ***	0.95 (0.94–0.98) ***
Animal-based food score	0.98 (0.94–1.02)	0.96 (0.92–1.01)	0.96 (0.92–1.01)	0.96 (0.92–1.01)

*Abbreviations*: *PDI* plant-based dietary index, *HR* hazard ratios, *95%CI0* 95% confidence intervals

Model 1: controlled for baseline ASM, gender and age

Model 2: controlled for baseline ASM, gender, age, residence, hunger in childhood, and education

Model 3: controlled for baseline ASM, gender, age, residence, hunger in childhood, education, BADL disability, IADL disability, regular physical examination, MMSE score, and current smoking, drinking, and exercise

Model 4: controlled for baseline ASM, gender, age residence, hunger in childhood, education, BADL disability, IADL disability, regular physical examination, MMSE score, and current smoking, drinking, and exercise, and history of disease

***: < 0.001

Further analysis of the associations of dietary scores and LMM across the age, gender and physical function subgroups were shown in Table 3. PDI presented a significant reduction of the risk of LMM in all subgroups except the male subpopulation, and the effect was more pronounced in the less eighty-year-old group (12% vs. 6%). Similarly, plant-based food scores showed the same trend as PDI, and animal-based food scores still did not show significant results in any particular subgroup.

**Table 3:** Associations of PDI with possible low muscle mass among age and physical function subpopulations

Indicators	PDI		Plant-based food score		Animal-based food score	
	HR (95%CI)	P-interaction	HR (95%CI)	P-interaction	HR (95%CI)	P-interaction
Age						
< 80 years	0.88 (0.83–0.93)***	0.05	0.88 (0.83–0.93)***	0.05	0.93 (0.85–1.02)	0.68
≥ 80 years	0.94 (0.91–0.97)***		0.96 (0.93–0.99)***		0.96 (0.91–1.00)	
Gender						
Men	0.98 (0.90–1.06)	0.29	0.92 (0.87–1.06)	0.74	1.03 (0.87–1.22)	0.23
Women	0.95 (0.93–0.97)***		0.96 (0.89–0.95)***		0.96 (0.92–1.00)	
BADL disability						
Yes	0.83 (0.71-0.94)***	0.09	0.85 (0.73-0.97)*	0.15	0.87 (0.71-1.06)	0.96
No	0.94 (0.91-0.96)**		0.93 (0.90-0.96)***		0.95 (0.90-1.00)	
IADL disability						
Yes	0.94 (0.90–0.98)**	0.96	0.95 (0.92–0.98)**	0.77	0.95 (0.90–1.01)	0.87
No	0.93 (0.89–0.96)***		0.92 (0.88–0.97)**		0.96 (0.89–1.04)	

*Abbreviations*: *PDI0* plant-based dietary index, *BADL* basic activity of daily living, *IADL* instrumental activity of daily living

^*^:< 0.05; **:< 0.01; ***:< 0.001

### Dietary groups and LMM

**Fig. 2:**
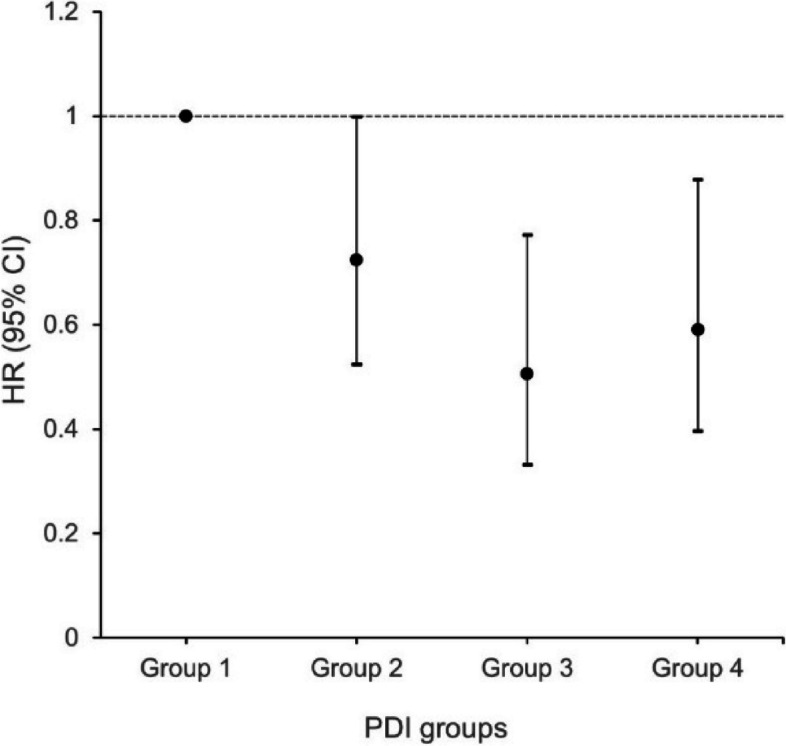
Hazard ratios (95% CI) of developing possible LMM by different PDI groups

Considering that the continuous dietary scores cannot distinguish well between plant-based and animal-based food consumption, a new classification of dietary groups considering both indicators was created. Compared to participants with the highest rate of LMM (Group 1), those with lower intake in both plant-based food and animal-based food (Group 2) had a 27.3% (HR:0.72, 95%CI: 0.52–0.99) lower risk of LMM during follow-up; the decreased risk of LMM was about 49.4% (HR:0.51, 95%CI: 0.33–0.77) for both plant-based food and animal-based food were higher intake (Group 3); and in those with high plant-based food intake and low animal-based food intake (Group 4), the risk of LMM was reduced by 40.9% (HR:0.59, 95%CI: 0.40–0.88) (Fig. 2 & Table S3).

Further subgroup analysis showed that both group 3 and group 4 could significantly reduce the risk of LMM in different age and women subgroups compared to group 1. In the physical function subgroups, different PDI groups presented different results. Compared to group 1, group 3 showed a significant reduction in the risk of LMM in both the BADL disability and IADL disability population, by 59.8% and 73.0%, respectively. The remaining groups did not show significant differences. However, for a relatively healthy function population, which means normal BADL function or normal IADL function, group 4 showed the best benefit in terms of LMM reduction. Both the detailed results are shown in Table 4.

**Table 4:** Associations of different plant-based dietary groups with possible low muscle mass among age and physical function subpopulations

Indicators	PDI groups			
	Group 1	Group 2	Group 3	Group 4
Age				
< 80 years	Ref	0.59 (0.28–1.24)	0.24 (0.08–0.63) **	0.33 (0.13–0.80) *
≥ 80 years	Ref	0.74 (0.48–1.15)	0.51 (0.30–0.86) *	0.55 (0.32–0.93) *
Gender				
Men	Ref	0.43 (0.13–1.47)	0.13 (0.02–1.07)	0.53 (0.15–1.88)
Women	Ref	0.77 (0.55–1.07)	0.55 (0.35–0.85) **	0.60 (0.39–0.91) *
BADL disability				
Yes	Ref	0.73 (0.49–1.07)	0.40 (0.24–0.66) ***	0.60 (0.03–1.26)
No	Ref	0.37 (0.07–1.95)	0.78 (0.16–3.78)	0.57 (0.35–0.90) *
IADL disability				
Yes	Ref	0.66 (0.35–1.23)	0.27 (0.11–0.61) **	0.57 (0.28–1.14)
No	Ref	0.75 (0.47–1.23)	0.57 (0.31–1.01)	0.51 (0.28–0.91) *

*Abbreviations*: Group 1: low plant food consumption and high animal food consumption; Group 2: low plant food consumption and low animal food consumption; Group 3: high plant food consumption and high animal food consumption; Group 4: high plant food consumption and low animal food consumption; *PDI* plant-based dietary index

^*^: < 0.05; **: < 0.01; ***: < 0.001

## Discussion

The PDI pattern was found to reduce the risk of decreased muscle mass in this prospective cohort study of 2771 Chinese older adults with normal muscle mass at baseline. Nevertheless, there were distinctions in the effects of plant-based and animal-based foods on different levels of physical function. The results of this study suggest that plant-based foods have a protective effect on muscle mass, but appropriate animal-based diets may have a preeminent impact on maintaining muscle mass in elderly individuals with poor physical function.

Previous studies have focused on the relationship between protein intake and muscle mass. A Japanese study showed a stronger correlation between animal protein intake and muscle mass, suggesting that animal-based foods favor the maintenance of muscle mass [6]. However, muscle mass was related to total protein intake rather than the type of protein in the Chinese population study [7, 22]. Our results also showed that animal-based foods were not significantly associated with muscle mass in older adults. A possible reason for this is that plant proteins accounted for a distinctively higher proportion of protein sources than animal proteins in the Chinese population, [7] contrary to the results of the Japanese study [6]. Therefore, higher PDI scores and plant-based food scores may suggest that more adequate plant protein sources have a better effect on maintaining muscle mass.

On the other hand, diet refers to more than just single nutrients, and unfortunately, to see that little has been involved in the evaluation of the relationship between more integrated, such as PDI, and muscle mass in the older population. According to a study from Hong Kong, a vegetable-fruit diet pattern and a snack beverage dairy pattern were associated with a lower prevalence of sarcopenia in older male Chinese [23]. For other groups, there is no difference in muscle mass between vegans and other vegetarians or omnivores who consume eggs or milk in healthy young adults [24]. In addition, supported by the Nurses' Health Study, a healthy plant-based diet was associated with a lower risk of frailty, while an unhealthy plant-based diet was the opposite [25]. Our study further approves the above research and finds that physical functional status may influence nutritional requirements, resulting in alterations to optimal dietary patterns in maintaining muscle mass.

For healthy older adults, a plant-based diet rich in antioxidant function and anti-inflammatory nutrients may help to maintain muscle mass hardy while meeting protein requirements simultaneously. First, legumes, nuts, and vegetable oils are rich in unsaturated fatty acids such as alpha-linolenic acid (ALA) and other nutrients that may help reduce plasma inflammatory cytokine levels and improve muscle inflammation associated with aging, thereby protecting muscle mass in older adults [26, 27]. A cross-sectional study also suggested that participants who adhered to a pro-inflammatory diet were more likely to develop sarcopenia [28]. In addition, vegetables and fruits are rich in nutrients, including polyphenols, antioxidant vitamins and plant proteins that reduce oxidative stress in muscles and positively result in mitochondrial modifications [22, 29]. Meanwhile, these nutrients can also favorably affect blood vessels during the aging process, ensuring the nutritional supply of muscles, and preventing a decline in muscle quality [30, 31]. Finally, PDI patterns protect nerve cells and reduce the development of neurodegenerative diseases [32], while neurotic plaque changes and motor neuron loss are among the essential physiological features of reduced muscle mass [1]. In contrast, animal-based foods, especially dietary patterns rich in red meat, may be associated with more cardiovascular and cerebrovascular diseases and disability [33]. Patients with metabolic diseases such as hypertension, and diabetes are also more likely to have reduced muscle mass. Therefore, the PDI pattern may be the optimal dietary pattern for older adults with normal physical function.

For people with physical dysfunction, high animal-based food intake appears to be more beneficial for maintaining muscle mass. One possible reason for this is that people with physical dysfunction tend to have a combination of multiple chronic diseases and lower physical activity levels, which lead to a higher protein intake requirement and a lower protein utilization [34, 35], so they need to consume higher amounts of protein from the diet. In addition, studies based on the characterization of the gut microbiota have also shown that the combination of plant and animal proteins is more beneficial for the eugenics of the gut microbiota and muscle protein synthesis than animal proteins alone [36]. Considering the low frequency of nutritional supplement use among the old group in low-income countries, animal-based foods are the best source of additional protein supplementation. Plant-based dietary patterns have a stronger correlation with muscle mass in women, and this may be related to gender-induced nutrient requirements [37] and hormone levels [38, 39], a trend that is the same in other studies [40].

Several studies have shown that the Chinese diet has shifted toward a more animal-based and less plant-based diet, including the elderly population, which may be related to economic development and income levels [41]. Our findings suggest that a high level of plant-based food intake is always beneficial to health, that PDI is more recommendable for healthy populations, and that the advertising of a rational diet should be enhanced to reduce the associated disease risk and economic burden.

Some strengths of the study included 1) the use of a nationally representative sample of older adults with relatively large sample sizes and the consideration of various confounding variables for adjustment; 2) we assessed the protective effect of a PDI pattern on muscle mass in older adults with limited physical function, which contributes to the robustness of our results and provides potential nutrient advice for older adults with limited physical function; 3) to our knowledge, we are one of the first studies to assess plant-based diets and muscle loss in a Chinese population.

Our study has several limitations. The ASM was calculated based on formula estimates rather than direct measurements by DXA, which may result in less accurate results for muscle mass despite the better accuracy of the prediction formula. Furthermore, diet data are based on self-report and may not accurately reflect the relationship between food intake and muscle mass due to memory bias. Moreover, our dietary questionnaire may not be sufficiently standardized, making replication difficult, although several studies have demonstrated the validity and reliability of non-quantitative food frequency questionnaires. Finally, health behaviors including physical exercise, smoking, and drinking are binary variables based on self-reported. According to ACSM guidelines, muscle quality had a significant relationship with the time and intensity of physical exercise and daily intake of alcohol and tobacco. However, our study did not collect those indicators. Additionally, we used activity frequency as a substitute for exercise time in sensitivity analysis, it may still have some bias in evaluating the impact of dietary patterns on muscle quality, but this will not affect the reliability of the conclusion.

In summary, this population-based study suggests that eating high levels of plant foods may be beneficial for maintaining muscle mass in older adults with or without functional dependency. Consequently, intervention ideas are provided for this population, which needs attention. There is a need for further longitudinal studies to confirm our findings and to reduce the burden of diminished quality of life and disease in older adults due to a reduction in muscle mass.

### Supplementary Information

**Additional file 1: Table S1.** Plant-based diet index scoring. **Table S2.** Baseline characteristics of participants by follow-up muscle mass. **Table S3.** Associations of different dietary scores and groups with possible Loss of Muscle Mass among whole sample, using the competing risk model. **Table S4.** Associations of different dietary scores and groups with possible Loss of Muscle Mass among whole sample.

## Data Availability

The data of this study are available to researchers upon reasonable request to corresponding author (Dr. Yao: yao.yao@bjmu.edu.cn).
