# Supplementary material for: Plant-based dietary pattern and low muscle mass: a nation-wide cohort analysis of Chinese older adults
Source: BMC Geriatr. 2023 Sep 16;23:569. doi: 10.1186/s12877-023-04265-7 (PMC10505314; doi:10.1186/s12877-023-04265-7)
Supplement: Supplementary file 1 — Additional file 1: Table S1. Plant-based diet index scoring. Table S2. Baseline characteristics of participants by follow-up muscle mass. Table S3. Associations of different dietary scores and groups with possible Loss of Muscle Mass among whole sample, using the competing risk model. Table S4. Associations of different dietary scores and groups with possible Loss of Muscle Mass among whole sample. [file 12877_2023_4265_MOESM1_ESM.docx]

# Supplement materials

Table S1 Plant-based diet index scoring

Table S2 Baseline characteristics of participants by follow-up muscle mass.

Table S3 Associations of different dietary scores and groups with possible Loss of Muscle Mass among whole sample, using the competing risk model.

Table S4 Associations of different dietary scores and groups with possible Loss of Muscle Mass among whole sample.

Table S1 Plant-based diet index scoring

| **Food category** | **Food** | **Frequency** | **PDI score** |
| --- | --- | --- | --- |
| Plant food | Whole grain | Yes | 5 |
|  |  | No | 1 |
|  | Vegetable oil | Yes | 5 |
|  |  | No | 1 |
|  | Fresh fruit | Almost everyday | 5 |
|  |  | Quite often | 4 |
|  |  | Occasionally | 2 |
|  |  | Rarely or never | 1 |
|  | Fresh vegetable | Almost everyday | 5 |
|  |  | Quite often | 4 |
|  |  | Occasionally | 2 |
|  |  | Rarely or never | 1 |
|  | Legume | Almost everyday | 5 |
|  |  | ≥1 time/week | 4 |
|  |  | ≥1 time/month | 3 |
|  |  | Occasionally | 2 |
|  |  | Rarely or never | 1 |
|  | Garlic | Almost everyday | 5 |
|  |  | ≥1 time/week | 4 |
|  |  | ≥1 time/month | 3 |
|  |  | Occasionally | 2 |
|  |  | Rarely or never | 1 |
|  | Nut | Almost everyday | 5 |
|  |  | ≥1 time/week | 4 |
|  |  | ≥1 time/month | 3 |
|  |  | Occasionally | 2 |
|  |  | Rarely or never | 1 |
|  | Tea | Almost everyday | 5 |
|  |  | ≥1 time/week | 4 |
|  |  | ≥1 time/month | 3 |
|  |  | Occasionally | 2 |
|  |  | Rarely or never | 1 |
|  | Mushroom or algae | Almost everyday | 5 |
|  |  | ≥1 time/week | 4 |
|  |  | ≥1 time/month | 3 |
|  |  | Occasionally | 2 |
|  |  | Rarely or never | 1 |
|  | Sugar | Almost everyday | 5 |
|  |  | ≥1 time/week | 4 |
|  |  | ≥1 time/month | 3 |
|  |  | Occasionally | 2 |
|  |  | Rarely or never | 1 |
|  | Salt-preserved vegetable | Almost everyday | 5 |
|  |  | ≥1 time/week | 4 |
|  |  | ≥1 time/month | 3 |
|  |  | Occasionally | 2 |
|  |  | Rarely or never | 1 |
| Animal food | Animal fat | Yes | 1 |
|  |  | No | 5 |
|  | Meat | Almost everyday | 1 |
|  |  | ≥1 time/week | 2 |
|  |  | ≥1 time/month | 3 |
|  |  | Occasionally | 4 |
|  |  | Rarely or never | 5 |
|  | Fish | Almost everyday | 1 |
|  |  | ≥1 time/week | 2 |
|  |  | ≥1 time/month | 3 |
|  |  | Occasionally | 4 |
|  |  | Rarely or never | 5 |
|  | Egg | Almost everyday | 1 |
|  |  | ≥1 time/month | 3 |
|  |  | Occasionally | 4 |
|  |  | Rarely or never | 5 |
|  | Dairy products | Almost everyday | 1 |
|  |  | ≥1 time/week | 2 |
|  |  | ≥1 time/month | 3 |
|  |  | Occasionally | 4 |
|  |  | Rarely or never | 5 |

Abbreviations: PDI: plant-based dietary index.

Table S2 Baseline characteristics of participants by follow-up muscle mass.

| **Characteristic** | **Normal muscle mass**  **(N=2537)** | **Loss of muscle mass**  **(N=234)** | ***p* Value** |
| --- | --- | --- | --- |
| Age, % |  |  | <0.001 |
| <80 years | 1,356 (53.45) | 51 (21.79) |  |
| ≥80 years | 1,181 (46.55) | 183 (78.21) |  |
| Gender, men, % | 1,450 (57.15) | 18 (7.69) | <0.001 |
| Education, % |  |  | <0.001 |
| 0 year | 1,157 (45.61) | 194 (82.91) |  |
| 1-6 years | 760 (29.96) | 25 (10.68) |  |
| >6 years | 620 (24.44) | 15 (6.41) |  |
| Residence, % |  |  | <0.001 |
| City | 304 (11.98) | 14 (5.98) |  |
| Town | 709 (27.95) | 65 (27.78) |  |
| Rural | 1,524 (60.07) | 155 (66.24) |  |
| Regular physical examination, % | 1,273 (50.18) | 94 (40.17) | 0.003 |
| Hunger in childhood, % | 1,700 (67.01) | 196 (83.76) | <0.001 |
| Exercise, % | 822 (32.4) | 32 (13.6) | <0.001 |
| Smoking, % | 560 (22.07) | 14 (5.98) | <0.001 |
| Drinking, % | 511 (20.14) | 23 (9.83) | <0.001 |
| MMSE, score | 26.83±4.91 | 24.59±6.77 | <0.001 |
| BADL disability, % | 172 (6.78) | 21 (8.97) | 0.207 |
| IADL disability, % | 1,076 (42.41) | 157 (67.09) | <0.001 |
| PDI, score | 48.68±5.72 | 45.92±5.74 | <0.001 |
| Plant-based food, score | 33.08±5.32 | 30.11±4.83 | <0.001 |
| Animal-based food, score | 15.60±3.07 | 15.82±3.01 | 0.313 |
| PDI groups, % |  |  | <0.001 |
| Group 1 | 493 (19.43) | 68 (29.06) |  |
| Group 2 | 709 (27.95) | 92 (39.32) |  |
| Group 3 | 733 (28.89) | 34 (14.53) |  |
| Group 4 | 602 (23.73) | 40 (17.09) |  |
| Hypertension, % | 889 (35.04) | 70 (29.91) | 0.015 |
| Diabetes, % | 135 (5.32) | 5 (2.14) | 0.033 |
| Heart disease, % | 325 (12.81) | 24 (10.26) | 0.260 |
| Stroke, cerebrovascular disease, % | 210 (8.28) | 12 (5.13) | 0.090 |
| Arthritis, % | 295 (11.63) | 23 (9.83) | 0.409 |
| Dyslipidemia, % | 103 (4.06) | 6 (2.56) | 0.260 |

Abbreviations: PDI: plant-based dietary index; Group 1: low plant food consumption and high animal food consumption; Group 2: low plant food consumption and low animal food consumption; Group 3: high plant food consumption and high animal food consumption; Group 4: high plant food consumption and low animal food consumption; LMM: loss of muscle mass; BADL: basic activity of daily living; IADL: instrumental activity of daily living; MMSE: Mini-Mental State Examination.

Table S3 Associations of different dietary scores and groups with possible Loss of Muscle Mass among whole sample, using the competing risk model

| Indicators | HR^a^ | 95% CI | *p* value |
| --- | --- | --- | --- |
| PDI, scores | 0.935 | 0.913-0.957 | <0.001 |
| Plant-based food, scores | 0.932 | 0.906-0.958 | <0.001 |
| Animal-based food scores | 0.950 | 0.910-0.992 | 0.020 |
| PDI groups |  |  |  |
| Group 1 | Ref |  |  |
| Group 2 | 0.702 | 0.505-0.978 | 0.037 |
| Group 3 | 0.475 | 0.312-0.724 | 0.001 |
| Group 4 | 0.525 | 0.345-0.798 | 0.002 |

Abbreviations: PDI: plant-based dietary index; Group 1: low plant food consumption and high animal food consumption; Group 2: low plant food consumption and low animal food consumption; Group 3: high plant food consumption and high animal food consumption; Group 4: high plant food consumption and low animal food consumption;

a: controlled for baseline ASM, gender, age residence, hunger in childhood, education, BADL disability, IADL disability, regular physical examination, current smoking and drinking, MMSE score, and history of disease.

Table S4 Associations of different dietary scores and groups with possible Loss of Muscle Mass among whole sample

| Indicators | HR^a^ | 95% CI | *p* value |
| --- | --- | --- | --- |
| PDI, scores | 0.933 | 0.909-0.958 | <0.001 |
| Plant-based food, scores | 0.964 | 0.942-0.986 | 0.002 |
| Animal-based food scores | 0.966 | 0.926-1.008 | 0.110 |
| PDI groups |  |  |  |
| Group 1 | Ref |  |  |
| Group 2 | 0.738 | 0.529-1.029 | 0.073 |
| Group 3 | 0.489 | 0.325-0.735 | 0.001 |
| Group 4 | 0.526 | 0.353-0.782 | 0.002 |

Abbreviations: PDI: plant-based dietary index; Group 1: low plant food consumption and high animal food consumption; Group 2: low plant food consumption and low animal food consumption; Group 3: high plant food consumption and high animal food consumption; Group 4: high plant food consumption and low animal food consumption;

a: controlled for baseline ASM, gender, age residence, hunger in childhood, education, BADL disability, IADL disability, regular physical examination, current smoking and drinking, frequency of individuals outdoor activities and social activities, MMSE score, and history of disease.
